# Intraocular Pressure Rise in Subjects with and without Glaucoma during Four Common Yoga Positions

**DOI:** 10.1371/journal.pone.0144505

**Published:** 2015-12-23

**Authors:** Jessica V. Jasien, Jost B. Jonas, C. Gustavo de Moraes, Robert Ritch

**Affiliations:** 1 Einhorn Clinical Research Center, New York Ear Eye and Ear Infirmary of Mount Sinai, New York, New York, United States of America; 2 Department of Ophthalmology, Medical Faculty Mannheim of the Ruprecht-Karls-University of Heidelberg, Seegartenklinik Heidelberg, Germany; 3 Department of Ophthalmology, Columbia University Medical Center, New York, New York, United States of America; Sun Yat-sen University, CHINA

## Abstract

**Purpose:**

To measure changes in intraocular pressure (IOP) in association with yoga exercises with a head-down position.

**Methods:**

The single Center, prospective, observational study included 10 subjects with primary open-angle glaucoma and 10 normal individuals, who performed the yoga exercises of Adho Mukha Svanasana, Uttanasana, Halasana and Viparita Karani for two minutes each. IOP was measured by pneumatonometry at baseline and during and after the exercises.

**Results:**

All yoga poses were associated with a significant (P<0.01) rise in IOP within one minute after assuming the yoga position. The highest IOP increase (P<0.01) was measured in the Adho Mukha Svanasana position (IOP increase from 17±3.2 mmHg to 28±3.8 mmHg in glaucoma patients; from 17±2.8 mmHg to 29±3.9 mmHg in normal individuals), followed by the Uttanasana position (17±3.9 mmHg to 27±3.4 mmHg (glaucoma patients) and from 18±2.5 mmHg to 26±3.6 mmHg normal individuals)), the Halasana position (18±2.8 mmHg to 24±3.5 mmHg (glaucoma patients); 18±2.7 mmHg to 22±3.4 mmHg (normal individuals)), and finally the Viparita Kirani position (17±4 mmHg to 21±3.6 mmHg (glaucoma patients); 17±2.8 to 21±2.4 mmHg (normal individuals)). IOP dropped back to baseline values within two minutes after returning to a sitting position. Overall, IOP rise was not significantly different between glaucoma and normal subjects (P = 0.813), all though glaucoma eyes tended to have measurements 2 mm Hg higher on average.

**Conclusions:**

Yoga exercises with head-down positions were associated with a rapid rise in IOP in glaucoma and healthy eyes. IOP returned to baseline values within 2 minutes. Future studies are warranted addressing whether yoga exercise associated IOP changes are associated with similar changes in cerebrospinal fluid pressure and whether they increase the risk of glaucoma progression.

**Trial Registration:**

ClinicalTrials.gov #NCT01915680

## Introduction

Glaucoma is the leading cause of irreversible blindness in the United States and can dramatically affect the quality of life for patients with moderate to severe visual loss. Primary open angle glaucoma is a progressive, chronic optic neuropathy characterized by a specific pattern of optic disc and visual field loss secondary to death of retinal ganglion calls and their axons and represents the final common pathway of multiple diseases which affect the eye. Elevated intraocular pressure (IOP) is the most common known risk factor for glaucomatous damage and, at the current time, the only modifiable one for which treatment has a proven effect on preventing or slowing the progress of the disease.

IOP increases on assuming a body position other than seated or upright. [1–7] A small variation can be detected when moving from the sitting to the recumbent position.[7–10] The increase in IOP is directly related to the inclination of the body toward the completely inverted position.[11, 12] IOP begins to rise upon assuming a head down position and with the body vertical, which results in doubling of the IOP[13], and IOP remains elevated as this position is maintained.[14–16] The extent of IOP fluctuations are correlated with the change of position based on angle (ninety degrees upright or inverted) and the length of time maintained. [5, 6, 11, 13, 15].

A relationship between posture-induced IOP fluctuations and visual field loss in glaucoma patients has been observed. Hirooka and Shiraga[5] reported that the greatest IOP fluctuation occurred in eyes with more severe glaucomatous optic nerve damage. The extent of increased IOP as measured in the horizontal supine position was associated with visual field damage in normal tension glaucoma in the horizontal position.[17]

Yoga has become a popular practice in the western world, and by 1998, an estimated 15 million American adults had performed yoga at least once. [16, 18–20] Elevation of IOP occurs during and following the sirsasana (headstand) posture, particularly in glaucoma patients.[16, 18, 20, 21] There was a uniform 2-fold increase in IOP in this position. [16, 20–22]

## Methods

**Fig 1:**
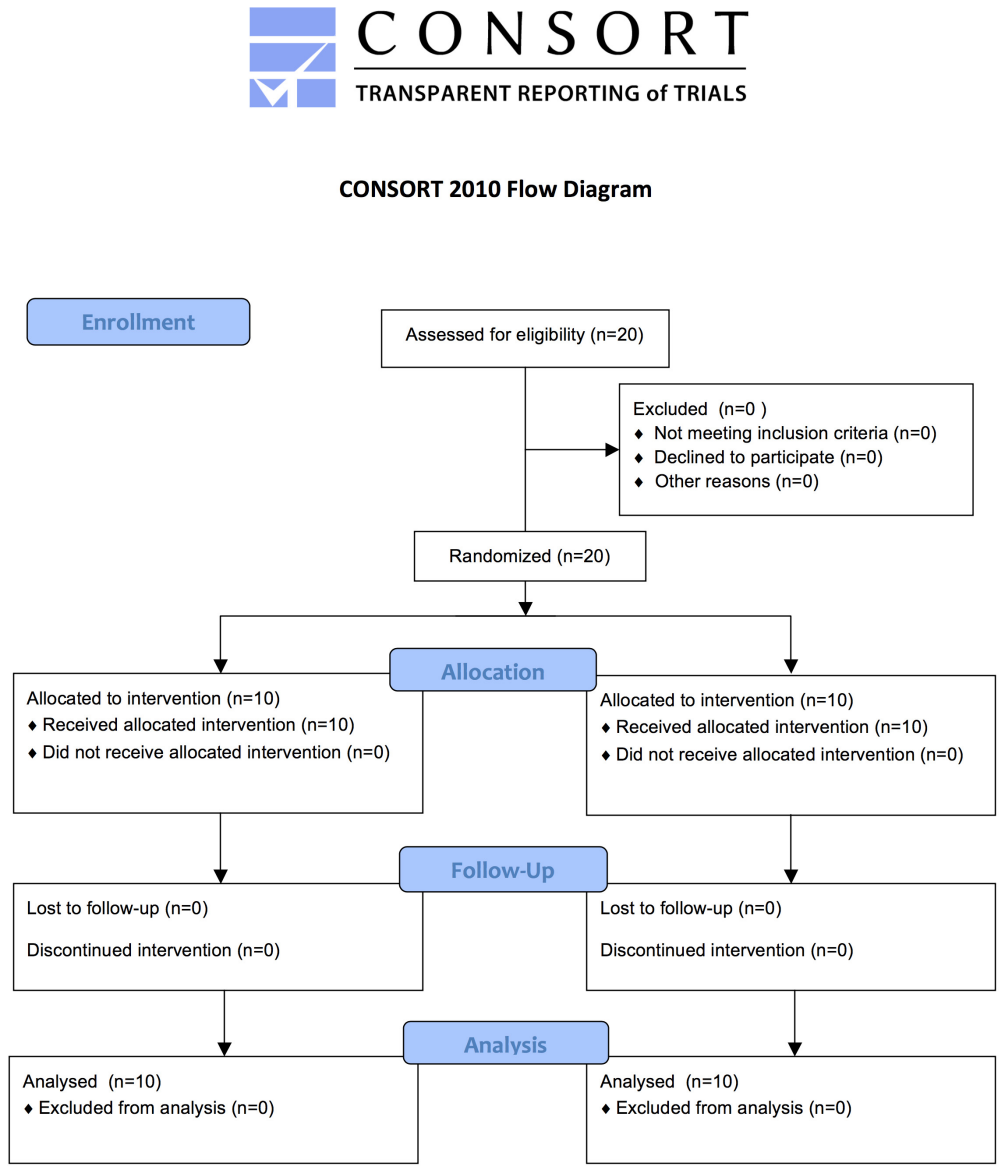
CONSORT Flow Diagram.

This was a prospective, observational study with a cohort of twenty subjects tested at the Einhorn Clinical Research Center, New York Eye and Ear Infirmary, New York, NY. The study was approved by the New York Eye and Ear Infirmary Institutional Review Board on March 12, 2013 and conformed to the tenets of the Declaration of Helsinki and approved under clinical trials registration identifier NCT01915680 on July 12, 2013. Written informed consent was obtained from all individuals. Participant recruitment started in June 2013 and was completed in September 2013; this time frame includes all study participant visits. The authors confirm that all ongoing and related trials for this study are registered. The delay in approval of clinical trial registration was due to study approval on clinicaltrials.gov. The CONSORT flow diagram is shown in Fig 1.

**Fig 2:**
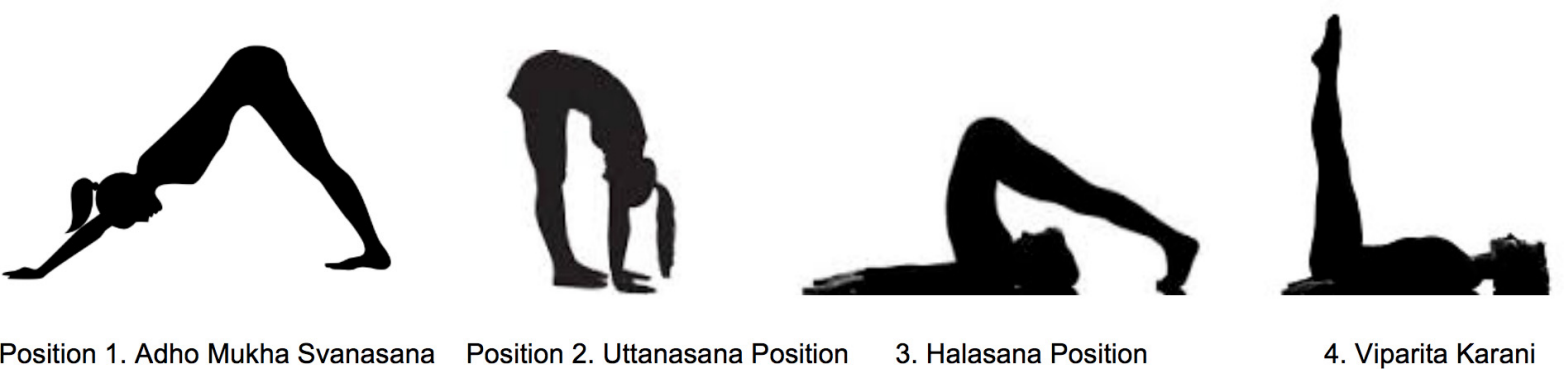
Scheme Illustrating the Various Yoga Positions.

Each participant assumed the four common yoga poses of Adho Mukha Svanasana, Uttanasana, Halasana and Viparita Karani in this respective order (Fig 2) within one hour. We measured the IOP of both eyes of the subjects prior to each pose in a seated position, immediately at the start of the pose, 2 minutes into the pose, immediately after assuming a seated position, and 10 minutes later in a seated position. IOP was measured using a Reichert Model 30 pneumatonometer, which was tested and calibrated using the calibration verifier before measuring each individual. Tetracaine was administered to each eye prior to IOP measurement using the calibrated pneumatonometer.

The Adho Mukha Svanasana pose, most commonly known as “downward facing dog”, was the first pose held by all subjects. Uttanasana, most commonly known as the “standard forward bend pose”, was then performed. The third position performed was Halasana, most commonly known as “plow pose”. Lastly, Viparita Karani, most commonly known as “legs up the wall pose” was performed to complete the sequence (Fig 2).[23]

The diagnosis of bilateral primary open-angle glaucoma was based on the presence of glaucomatous optic nerve head changes and visual field loss, gonioscopically open anterior chamber angles and no identifiable secondary cause of glaucoma. Optic nerve head changes as assessed on stereoscopic photographs included focal or diffuse thinning of the neuroretinal rim, focal or diffuse loss of the retinal nerve fiber layer, or an inter-eye difference in the vertical cup-to-disc diameter ratio of >0.2 not explained by inter-eye differences in optic disc size. Criteria for glaucomatous visual field loss, tested by the 24–2 Swedish Interactive Thresholding Algorithm (SITA) (SITA-SAP, Humphrey Visual Field Analyzer; Carl Zeiss Meditec, Inc., Dublin, CA) were a glaucoma hemifield test result outside normal limits on at least two consecutive reliable examinations or the presence of at least three contiguous test points on the pattern standard deviation plot with *P* <1%, and with at least one of *P* <0.5%, not including points at the edge of the field or those directly above and below the blind spot. All 24–2 visual fields had to have reliability indices of <25% for fixation losses, false-positive responses and false-negative responses.

Statistical analysis was carried out with commercially available software (STATA, version 12; StataCorp LP, College Station, TX). All continuous variables, except visual field mean deviation (MD), followed a Gaussian distribution based on visual inspection of Q-Q plots and the Shapiro-W test (all P>0.10). Therefore, all descriptive statistics are presented with mean ± standard deviation unless otherwise specified. To explore adequacy of a linear model when testing the relationship between IOP and the set of predictors, we plotted the histograms of residuals and the relationship between fitted values and residuals to test for homoscedasticity. IOP changes for each subject and each yoga position were tested with the mixed-effects linear models (MELM). Multilevel MELM analysis was performed at three levels: 1) position type; 2) diagnostic groups (glaucoma vs. healthy); and 3) each subject at different time points.

For the multilevel MELM results interpretation, the interaction term ‘Pose*Time’ provides a test for differential IOP over time due to the different poses investigated; the interaction term ‘Group*Pose’ provides a test for differences in average IOP across poses due to diagnostic groups (i.e.: glaucoma vs normal); and the interaction term ‘Group*Time*Pose’ provides a test for differential IOP over time for each pose due to diagnostic groups.

**Fig 3:**
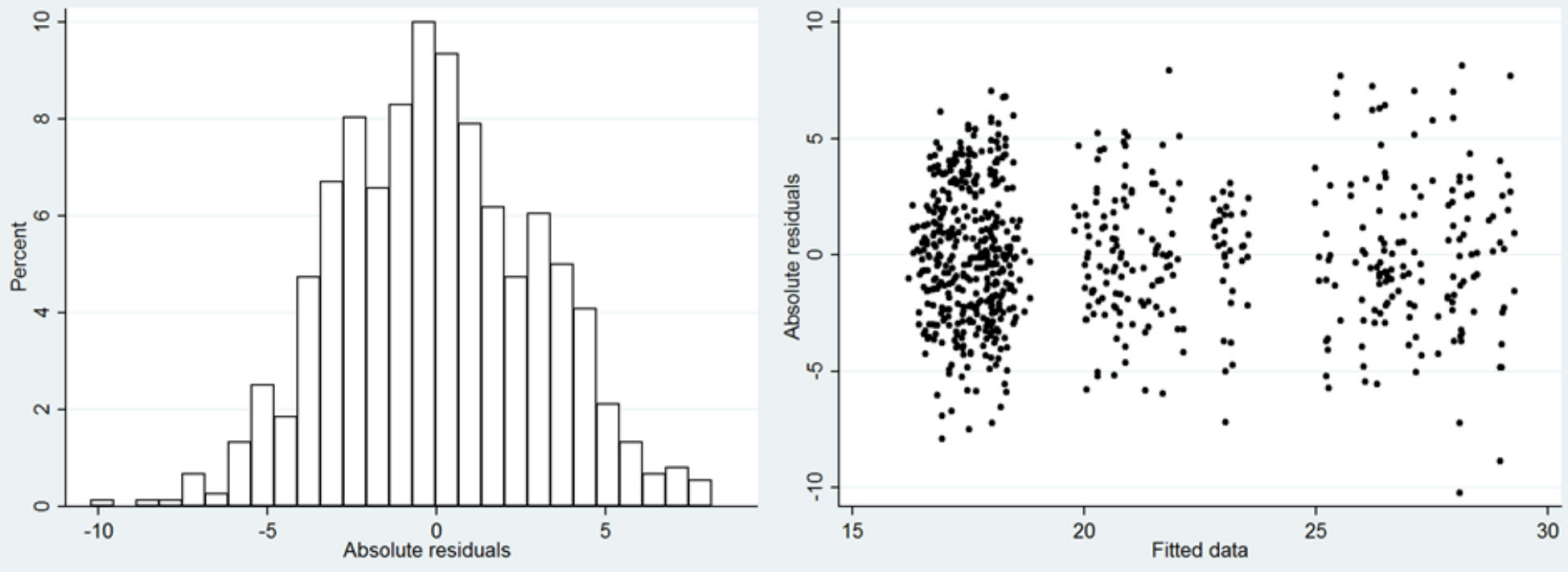
Histogram and test for homoscedasticity of the mixed effects linear model.

The model was fitted with fixed coefficients (fixed effect) of participants baseline age (years), time (prior to each pose in a seated position, immediately at the start of the pose, 2 minutes into the pose, immediately after assuming a seated position, and 10 minutes later in a seated position), BMI (kg/m^2^), diagnostic group (glaucoma or control), and Yoga pose. The random coefficient (random effect) relates to the subject (i.e.: each eye nested within subject) to detect the effect of posture changes over time. The inclusion of random eye effects accounts for the non-independence of the 2 eyes from the same subject. The covariance structure at each level was treated as compound symmetric (exchangeable). Glaucoma diagnosis, body mass index, and age were entered as predictors. Since the categorical predictor ‘Diagnostic group (glaucoma vs. normal) is highly correlated with the variable ‘MD’, we chose to remove the variable ‘MD’ from the analysis of predictors associated with IOP change. After fitting the MELM, we plotted histograms of the residuals to evaluate whether the residuals are consistent with normally distributed errors (Fig 3).

Finally, we performed an analysis of contrasts with baseline IOP values for each yoga pose against each other to check against ‘carry-over’ (or sequence) effects, that is, whether the fact that subjects did not assume the poses in random order could have affected our results. In addition, this analysis provides estimates and contrasts versus baseline for each yoga pose. The same type of analysis was performed for estimates and contrasts involving comparisons among poses, diagnostic groups, and time. Statistical significance was declared at the 0.05 level.

## Results

The study included 10 subjects (9 women; median age: 62 years ± 15.5 years) with primary open-angle glaucoma and 10 healthy individuals (8 women; median age: 36 years ± 12.4 years) (Table 1). The difference in age between both groups was statistically significant (*P*<0.001). The median of the mean visual field defect of the glaucoma patients was -9.49 dB (interquartile rage: -1.67 dB to -21.13 dB). Regressions diagnostics of the MELM are shown in Fig 2. The normal distribution of the residuals and the spread of the residuals relative to fitted values (homoscedasticity) suggest adequacy of the model, the results of which are described below.

**Table 1:** Demographic and Baseline Characteristics of Study Participants.

Characteristic	Primary Open-Angle Glaucoma Participants	Healthy Participants
**Number of Participants**	10	10
**Females (%)**	90	80
**Mean age in years**	62 ± 15.5	36 ± 12.4
**Mean BMI in kg (m** ^**2**^ **)**	22.1	23.8

**Fig 4:**
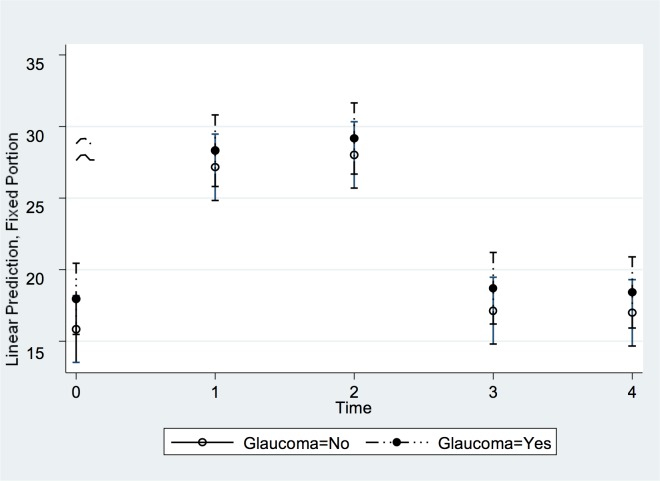
Least squares means and mean values of the covariates of the changes in IOP in the Adho Mukha Svanasana position over time. The X axis represents each time point an IOP measurement was taken (0 = Baseline seated; 1 = Immediate position; 2 = 2 minutes position; 3 = Post position seated; and 4 = 10 minutes post position seated). The Y axis represents the IOP in mmHg after adjusting for the covariates. Subjects with glaucoma diagnosis are depicted ‘Glaucoma = 1’; controls are ‘Glaucoma = 0’.

Within both groups, IOP increased significantly for all 4 yoga positions (repeated-measures ANOVA; all *P*<0.001). The Adho Mukha Svanasana position was associated with the highest IOP increase (*P*<0.01) (Fig 4). IOP increased from 16.7 ± 3.0 mmHg (median: 17 mmHg; range: 12, 23) to 28.5 ± 3.8 mmHg (median: 28 mmHg; range: 19, 38) at two minutes of holding the pose. The maximum increase in IOP, measured immediately after taking the pose or at two minutes of holding the pose, did not differ significantly (*P* = 0.57) between the control group (12.6 ± 3.5 mmHg; median: 12 mmHg; range: 8 mmHg, 19 mmHg) and the glaucoma group (11.6 ± 3.2 mmHg; median: 10 mmHg; range: 6 mmHg, 17 mmHg) (Table 2). Related to the baseline values, the IOP increased by 79 ± 31% (median: 75%; range: 38%, 158%) in the control group and by 72 ± 29% (median: 61%; range: 40%, 126%) in the glaucoma group. All eyes showed an increase in IOP during the pose.

**Table 2:** Interquartile Range, Mean and Standard Deviations (SD) of Intraocular Pressure in Normal Individuals and Glaucoma patients for Each Yoga Position and Time Point.

		Baseline Seated	Immediate Position	Two Minutes Position	Post Position Seated	Ten Minutes Post Position Seated	Maximal Difference in Intraocular Pressure between Baseline and Holding the Pose	
Adho Mukha Svanasana							mmHg	%
Normals	Mean ± SD	16.6 ± 2.8	28.1 ± 4.2	28.8 ± 3.9	17.9 ± 2.6	19.0 ± 2.5	12.6 ± 3.5	79 ± 31%
Glaucoma	Mean ± SD	16.9 ± 3.2	27.3 ± 4.3	28.1 ± 3.8	17.6 ± 3.7	17.3 ± 3.8	11.6 ± 3.2	72 ± 29%
Normals	25%, 50%, 75%	14, 17, 18	25, 27, 32	27, 28, 32	17, 18, 20	17, 18, 20	
Glaucoma	25%, 50%, 75%	14, 17, 19	26, 28, 30	27, 28, 31	15, 19, 20	15, 18, 20	
Uttanasana								
Normals	Mean ± SD	18.0 ± 2.5	25.3 ± 3.8	26.1 ± 3.6	18.1 ± 3.1	18.3 ± 3.0	8.4 ± 3.4	48 ± 21%
Glaucoma	Mean ± SD	17.3 ± 3.8	26.6 ± 3.2	26.5 ± 3.0	18.1 ± 4.4	17.5 ± 3.3	9.8 ± 2.7	61 ± 26%
Normals	25%, 50%, 75%	17, 18, 20	22, 26, 29	23, 26, 28	17, 18, 20	16, 18, 20	
Glaucoma	25%, 50%, 75%	15, 18, 20	26, 27, 29	26, 27, 28	14, 19, 21	15, 18, 20	
Halasana								
Normals	Mean ± SD	17.8 ± 2.7	21.5 ± 2.7	21.9 ± 3.4	16.8 ± 2.0	16.5 ± 2.0	4.7 ± 3.1	28 ± 19%
Glaucoma	Mean ± SD	18.2 ± 2.6	23.1 ± 2.6	23.1 ± 3.6	18.4 ± 2.9	18.1 ± 3.5	5.7 ± 2.6	33 ± 17%
Normals	25%, 50%, 75%	16, 18, 20	20, 22, 23	20, 22, 24	16, 17, 18	15, 16, 18	
Glaucoma	25%, 50%, 75%	16, 19, 20	21, 23, 25	21, 22, 24	17, 18, 20	16, 18, 21	
Viparita Karani								
Normals	Mean ± SD	17.2 ± 2.8	20.7 ± 2.4	20.1 ± 2.6	17.0 ± 3.2	16.6 ± 2.2	4.0 ± 2.2	25 ± 16%
Glaucoma	Mean ± SD	17.6 ± 3.8	21.0 ± 3.4	20.4 ± 3.5	17.8 ± 3.2	17.2 ± 3.2	3.7 ± 1.5	23 ± 12%
Normals	25%, 50%, 75%	16, 17, 19	19, 21, 22	18, 20, 22	15, 16, 18	15, 16, 18	
Glaucoma	25%, 50%, 75%	15, 18, 21	19, 22, 23	19, 21, 22	16, 17, 20	15, 17, 19	

**Fig 5:**
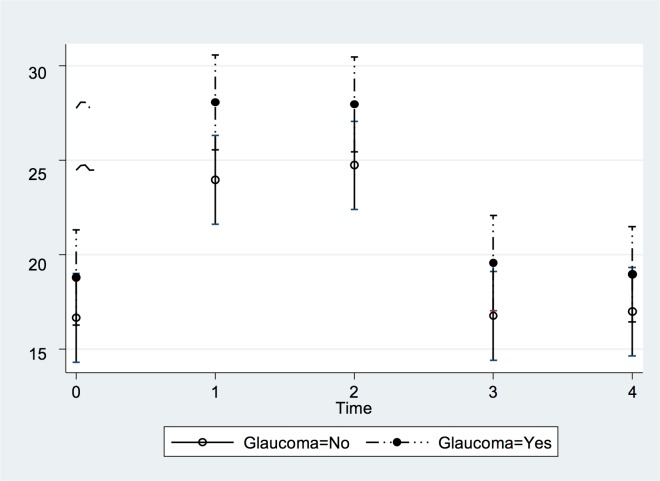
Least squares means and mean values of the covariates of the changes in IOP in the Uttanasana position over time. The X axis represents each time point an IOP measurement was taken (0 = Baseline seated; 1 = Immediate position; 2 = 2 minutes position; 3 = Post position seated; and 4 = 10 minutes post position seated). The Y axis represents the IOP in mmHg after adjusting for the covariates. Subjects with glaucoma diagnosis are depicted ‘Glaucoma = 1’; controls are ‘Glaucoma = 0’.

During the Uttanasana pose, IOP increased from 17.7 ± 3.1 mmHg (median: 18 mmHg; range: 12, 25) to 26.2 ± 3.3 mmHg (median: 27 mmHg; range: 19, 33) at two minutes of holding the pose (Fig 5). The maximum increase in IOP, measured immediately after taking the pose or at two minutes of holding the pose, did not differ significantly (*P* = 0.16) between the control group (8.4 ± 3.4 mmHg; median: 9 mmHg; range: 2 mmHg, 15 mmHg) and the glaucoma group (9.8 ± 2.7 mmHg; median: 9 mmHg; range: 6 mmHg, 15 mmHg) (Table 2). Related to the baseline values, the IOP increased by 48 ± 21% (median: 49%; range: 7%, 86%) in the control group and by 61 ± 26% (median: 48%; range: 24%, 107%) in the glaucoma group. All eyes showed an increase in IOP during the pose.

**Fig 6:**
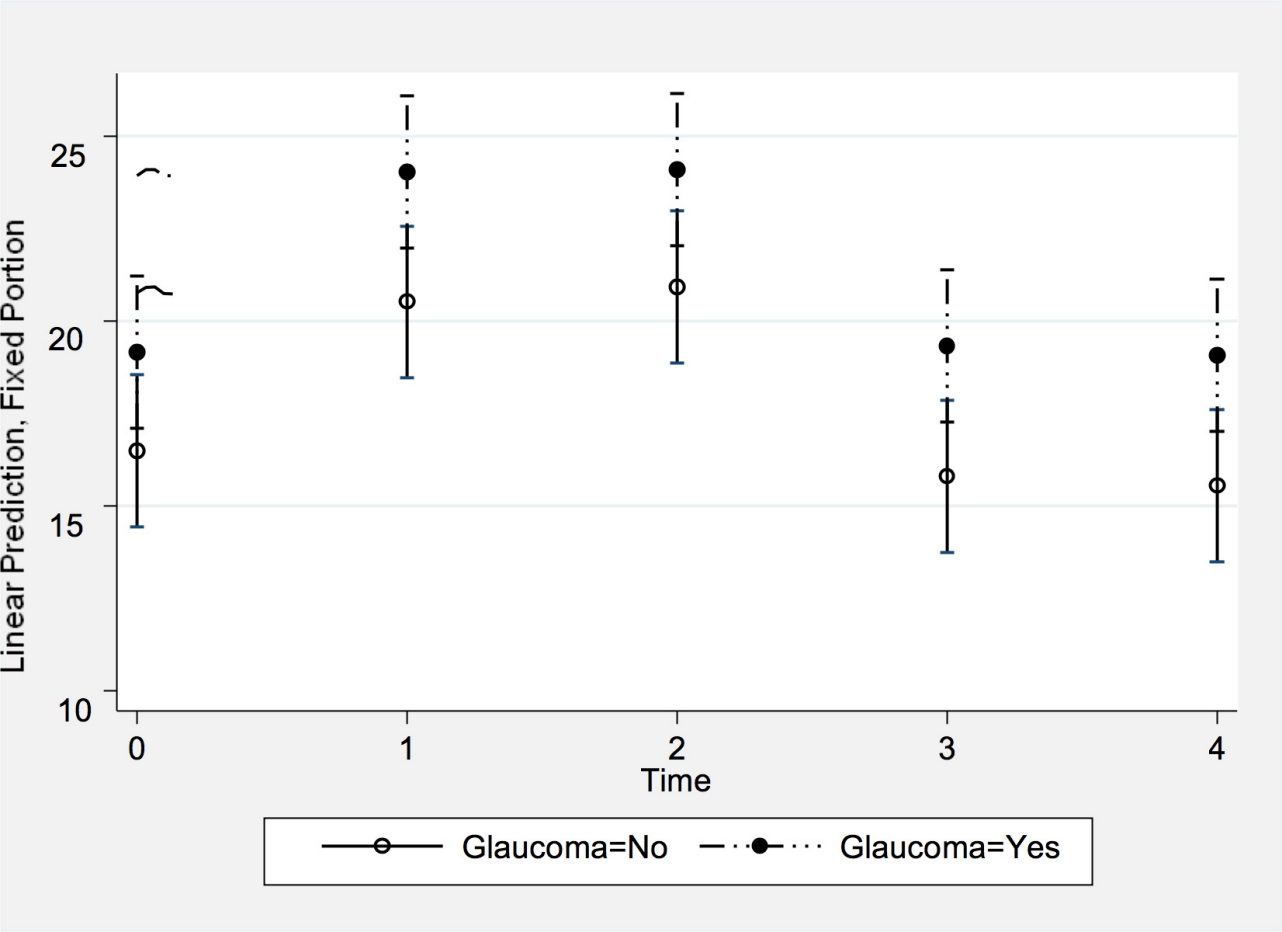
Least squares means and mean values of the covariates of the changes in IOP in the Halasana position over time. The X axis represents each time point an IOP measurement was taken (0 = Baseline seated; 1 = Immediate position; 2 = 2 minutes position; 3 = Post position seated; and 4 = 10 minutes post position seated). The Y axis represents the IOP in mmHg after adjusting for the covariates. Subjects with glaucoma diagnosis are depicted ‘Glaucoma = 1’; controls are ‘Glaucoma = 0’.

During the Halasana position, IOP increased from 18.0 ± 2.6 mmHg (median: 18 mmHg; range: 13, 23) to 22.5 ± 3.5 mmHg (median: 22 mmHg; range: 14, 31) at two minutes of holding the pose (Fig 6). The maximum increase in IOP, measured immediately after taking the pose or at two minutes of holding the pose, did not differ significantly (*P* = 0.29) between the control group (4.7 ± 3.1 mmHg; median: 5 mmHg; range: -2 mmHg, 10 mmHg) and the glaucoma group (5.7 ± 2.6 mmHg; median: 6 mmHg; range: 0 mmHg, 10 mmHg) (Table 2). Related to the baseline values, the IOP increased by 28 ± 19% (median: 31%; range: -8%, 54%) in the control group and by 33 ± 17% (median: 35%; range: 0%, 67%) in the glaucoma group. All but one eye in the control group showed an increase in IOP during the pose.

**Fig 7:**
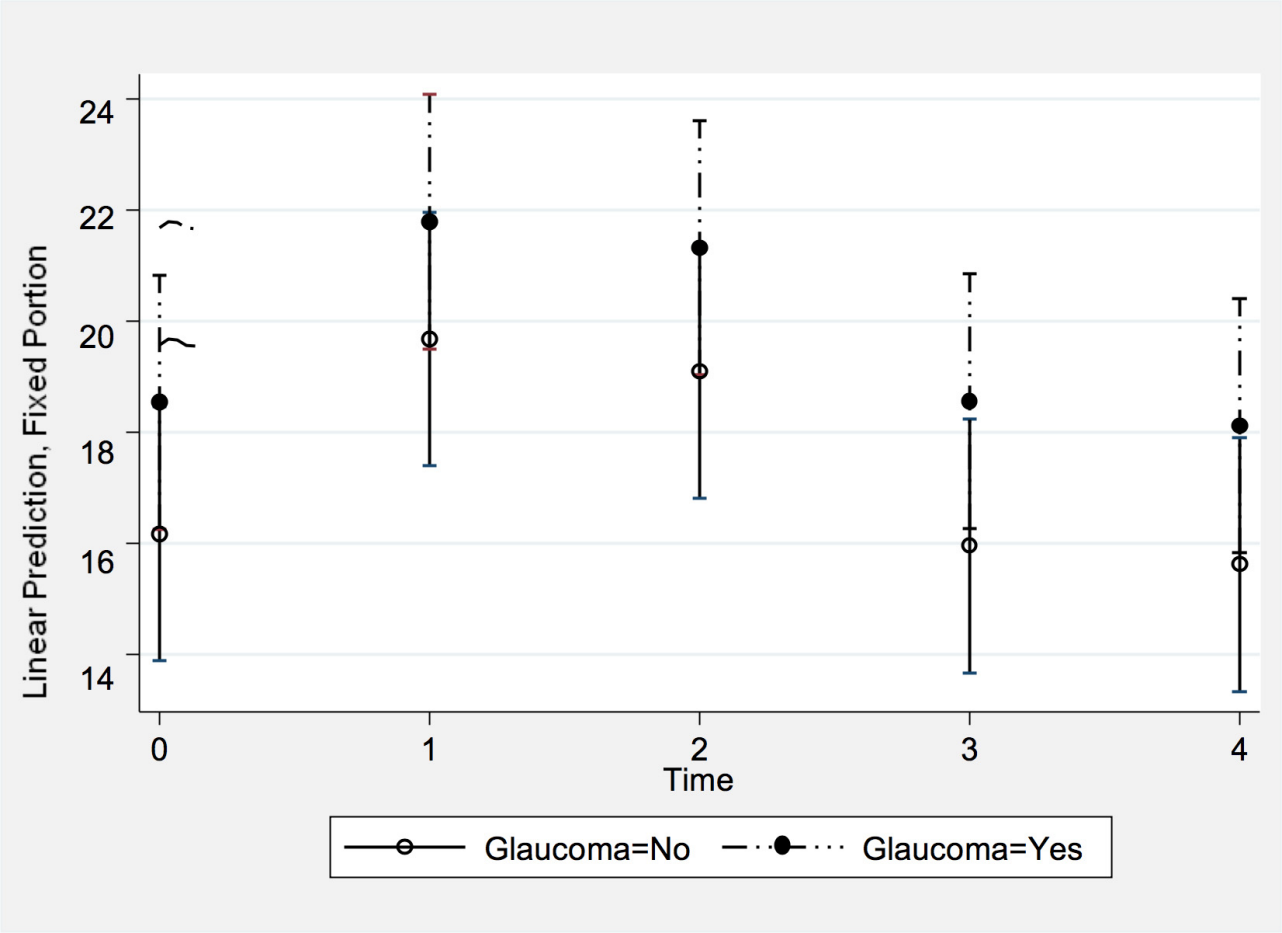
Least squares means and mean values of the covariates of the changes in IOP in the Viparita Karani position over time. The X axis represents each time point an IOP measurement was taken (0 = Baseline seated; 1 = Immediate position; 2 = 2 minutes position; 3 = Post position seated; and 4 = 10 minutes post position seated). The Y axis represents the IOP in mmHg after adjusting for the covariates. Subjects with glaucoma diagnosis are depicted ‘Glaucoma = 1’; controls are ‘Glaucoma = 0’.

During the Viparita Kirani position, IOP increased from 17.4 ± 3.3 mmHg (median: 17 mmHg; range: 12, 26) to 20.2 ± 3.1 mmHg (median: 20 mmHg; range: 13, 27) at two minutes of holding the pose (Fig 7). The maximum increase in IOP, measured immediately after taking the pose or at two minutes of holding the pose, did not differ significantly (*P* = 0.65) between the control group (4.0 ± 2.2 mmHg; median: 4 mmHg; range: 1 mmHg, 9 mmHg) and the glaucoma group (3.7 ± 1.5 mmHg; median: 3 mmHg; range: 1 mmHg, 6 mmHg) (Table 2). Related to the baseline values, the IOP increased by 25 ± 16% (median: 25%; range: 3%, 60%) in the control group and by 23 ± 12% (median: 21%; range: 2%, 46%) in the glaucoma group. All eyes showed an increase in IOP during the pose.

Similar results were obtained if only one eye per individual was included into the statistical analysis. Multilevel MELM results (Table 3, interaction ‘Pose*Time’) showed that the increase in IOP from baseline was significant (*P*<0.001) for all yoga poses in the glaucoma group as well as in the control group immediately at the start of the pose and 2 minutes into the pose. The IOP then tended to return to baseline values immediately after assuming a seated position and 10 minutes later in a seated position (P>0.05 in most cases). Nonetheless, overall changes in IOP values between the glaucoma group and the control group were not statistically significant (P = 0.813). The interaction term ‘Group*Time*Pose’ reveals that the glaucoma group had higher IOP measurements at the start and 2 minutes into the Uttanasana pose when compared to controls (P = 0.017 and 0.098, respectively).

**Table 3:** Analysis of a Mixed-Effects Regression between the Change in Intraocular Pressure and Various Parameters during Yoga Exercises with Head-Down Positions.

IOP	Coef.	95% Conf. Interval		P-value
**Time**	0.28	-0.05	0.61	0.095
**Age**	-0.01	-0.06	0.03	0.545
**BMI**	-0.01	-0.16	0.13	0.86
**Group: Glaucoma**	0.32	-2.35	2.99	0.813
**Pose Reference:**				
**Adho Mukha Svasana (1)**				
**Uttanasana (2)**	1.13	-1.27	3.52	0.357
**Halasana (3)**	0.59	-1.87	3.05	0.637
**Viparita Karani (4)**	0.3	-2.09	2.69	0.806
Group*Pose				
**1 2**	-0.68	-4.16	2.8	0.701
**1 3**	0.74	-2.79	4.26	0.682
**1 4**	0.44	-2.99	3.88	0.8
**Time Reference:**				
**Baseline (0)**				
**1**	11.02	9.98	12.06	**<0.001**
**2**	11.61	10.62	12.61	**<0.001**
**3**	0.43	-0.61	1.47	0.415
**4**	0			
Group*Time				
**1 1**	-0.94	-2.63	0.75	0.276
**1 2**	-0.98	-2.72	0.76	0.268
**1 3**	-0.53	-2.34	1.29	0.571
**1 4**	-0.68	-2.6	1.24	0.487
Pose*Time				
**2 1**	-4	-5.64	-2.36	**<0.001**
**2 2**	-4.1	-5.79	-2.41	**<0.001**
**2 3**	-1.18	-2.94	0.59	0.192
**2 4**	-0.8	-2.67	1.07	0.401
**3 1**	-7.22	-8.9	-5.53	**<0.001**
**3 2**	-7.73	-9.47	-5.99	**<0.001**
**3 3**	-1.91	-3.73	-0.1	0.039
**3 4**	-2.07	-3.99	-0.15	0.035
**4 1**	-7.78	-9.42	-6.13	**<0.001**
**4 2**	-9.25	-10.94	-7.56	**<0.001**
**4 3**	-1.48	-3.24	0.29	0.102
**4 4**	-1.68	-3.54	0.19	0.079
Group*Pose*Time				
**1 2 1**	2.92	0.53	5.3	0.017
**1 2 2**	2.07	-0.38	4.53	0.098
**1 2 3**	1.2	-1.36	3.77	0.358
**1 2 4**	0.52	-2.19	3.24	0.706
**1 3 1**	1.77	-0.65	4.19	0.151
**1 3 2**	1.48	-1.01	3.97	0.243
**1 3 3**	1.39	-1.21	3.99	0.296
**1 3 4**	1.54	-1.21	4.29	0.272
**1 4 1**	0.79	-1.57	3.14	0.512
**1 4 2**	0.83	-1.59	3.25	0.502
**1 4 3**	0.88	-1.66	3.41	0.498
**1 4 4**	0.81	-1.87	3.48	0.555

*P*<0.01 denotes statistical significance.

**Fig 8:**
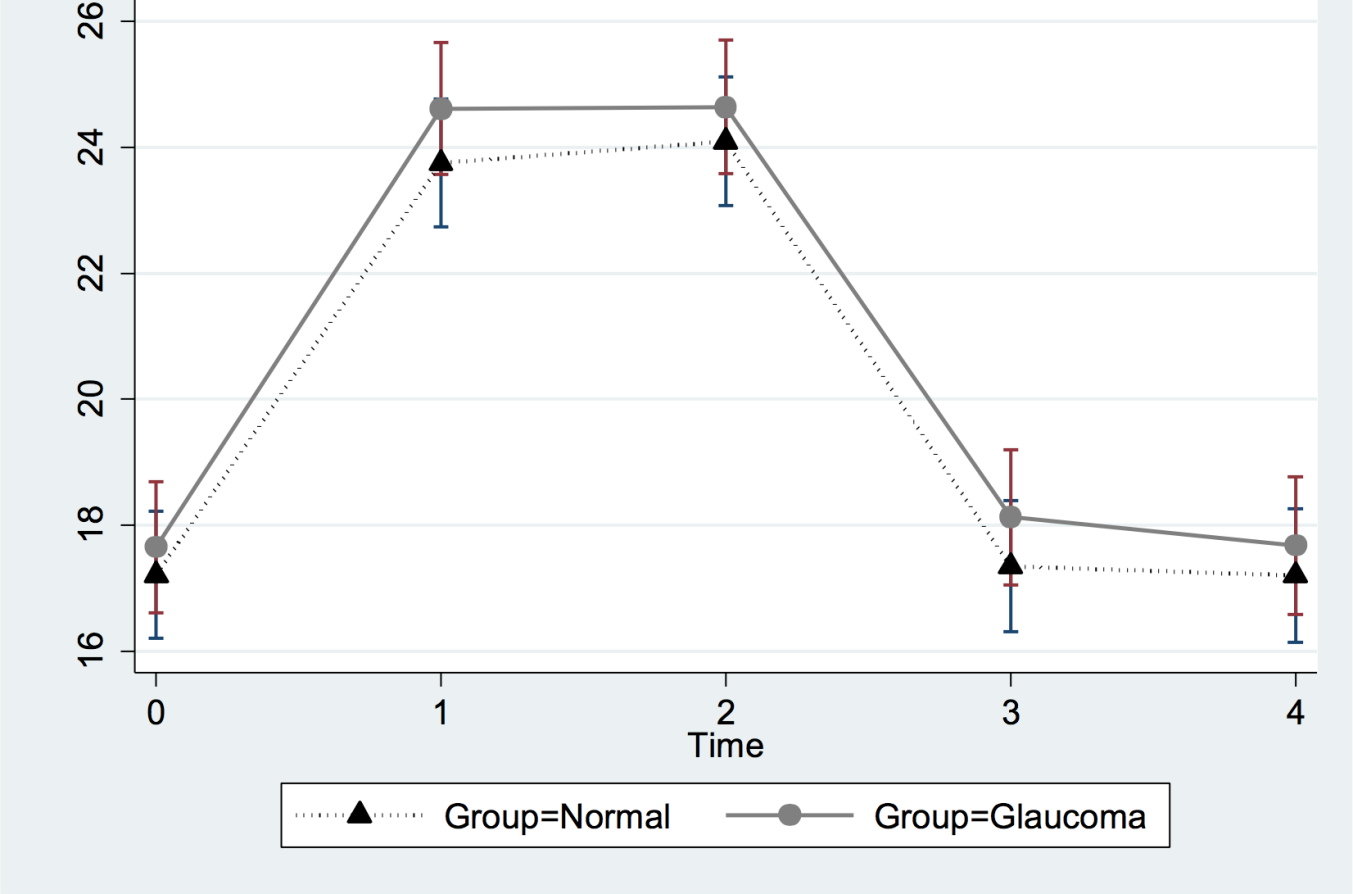
Predictive Margins of Diagnostic Groups versus Time with 95% Confidence Intervals.

**Fig 9:**
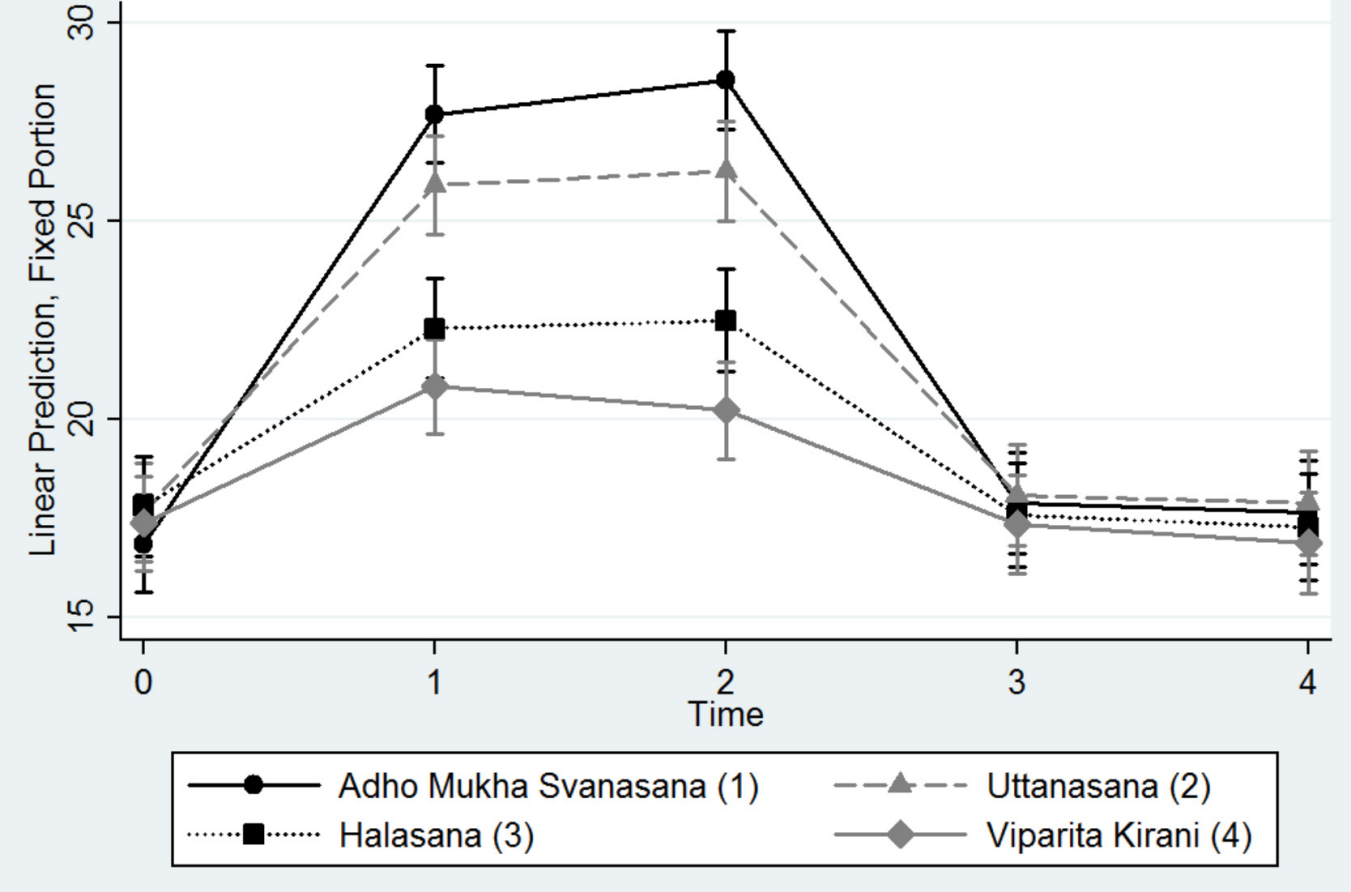
Predictive Margins of Yoga Pose versus Time with 95% Confidence Intervals.

Tables 4 and 5 and Figs 8 and 9 show the results of the analysis of margins and contrasts for the predictors pose, diagnostic group, and time. Even though IOP changed at different time points (significantly when comparing immediately at the start of the pose and 2 minutes into the pose vs. baseline), these changes did not differ significantly between glaucoma and healthy eyes (although glaucoma eyes had IOPs 1 to 2 mmHg higher on average). This is also shown in Fig 8. Table 5 shows that poses Adho Mukha Svanasana and Uttanasana on average led to higher IOP increases than the other two and suggest that no carry-over effect occurred even though the poses were not performed in a random sequence. This is also shown in Fig 9. Lastly, body mass index and age were not significantly associated with IOP changes for any yoga position (Table 3).

**Table 4:** Analysis of margins and contrasts for the predictors pose, diagnostic group, and time.

IOP						
Group@Time	Contrast	Std. Err.	z	P>|z|	[95% Conf. Interval]	
**(1 vs base) 0**	0.4479324	0.8388864	0.53	0.593	-1.196255	2.092119
**(1 vs base) 1**	0.8784879	0.8410353	1.04	0.296	-0.769911	2.526887
**(1 vs base) 2**	0.5632102	0.8474495	0.66	0.506	-1.09776	2.224181
**(1 vs base) 3**	0.7889046	0.8580332	0.92	0.358	-0.8928095	2.470619
**(1 vs base) 4**	0.4847379	0.8726347	0.56	0.579	-1.225595	2.195071

**Table 5:** Analysis of margins and contrasts for the predictors pose, diagnostic group, and pose*time.

IOP- Pose@Time	Contrast	Std. Err.	z	P>|z|	[95% Conf. Interval]	
**(2 vs base) 0**	0.7847222	0.887311	0.88	0.376	-0.9543754	2.52382
**(2 vs base) 1**	-1.756944	0.8913703	-1.97	0.049	-3.5033998	-0.0098908
**(2 vs base) 2**	-2.279167	0.9034386	-2.52	0.012	-4.049874	-0.5084595
**(2 vs base) 3**	0.2111111	0.9232021	0.23	0.819	-1.598332	2.020554
**(2 vs base) 4**	0.2458333	0.9501805	0.26	0.796	-1.616486	2.108153
**(3 vs base) 0**	0.9608382	0.8990916	1.07	0.285	-0.8013489	2.723025
**(3 vs base) 1**	-5.369717	0.9032031	-5.95	0	-7.139963	-3.599472
**(3 vs base) 2**	-6.02944	0.9154268	-6.59	0	-7.823643	-4.235236
**(3 vs base) 3**	-0.2599951	0.9354448	-0.28	0.781	-2.093433	1.573443
**(3 vs base) 4**	-0.3377729	0.9627709	-0.35	0.726	-2.223769	1.549223
**(4 vs base) 0**	0.5223753	0.8759818	0.6	0.551	-1.194518	2.239268
**(4 vs base) 1**	-6.85818	0.8799854	-7.79	0	-8.58292	-5.133441
**(4 vs base) 2**	-8.312347	0.8918883	-9.32	0	-10.06042	-6.564278
**(4 vs base) 3**	-0.5151247	0.911381	-0.57	0.572	-2.301399	1.271149
**(4 vs base) 4**	-0.7498469	0.9379905	-0.8	0.424	-2.588275	1.088581

## Discussion

We tested the hypothesis that changes in body position during yoga lead to changes in IOP among both healthy and POAG subjects. We confirmed the hypothesis and observed that the position-associated IOP changes occurred immediately within one to two minutes after assuming the position, returning to values close to baseline after assuming a seated position and 10 minutes later in a seated position. Glaucoma diagnosis did not show a significant effect on IOP increase and poses Adho Mukha Svanasana and Uttanasana were associated with greater IOP elevation.

Both normal and glaucoma subjects showed a rise in IOP in all four yoga positions. Independent of the position, the rise ranged between 6 mmHg and 11 mmHg. It occurred within one minute after assuming the body position of the yoga exercise, and the IOP returned to the baseline values within two minutes after again being seated, with no further significant changes thereafter. The results suggest that all individuals experience an acute elevation in IOP immediately after assuming certain common yoga positions. This rise in IOP lasts as long as the exercise takes place, and the IOP returns to the baseline values shortly after sitting. The duration of the yoga pose was two minutes.

Our results agree with those of previous studies and case reports which tested only the headstand position and which showed a marked two-fold rise in IOP.[16, 21, 22] Our study extends those findings to show that other yoga exercises with a head down position can lead to a rapid and profound elevation in IOP. The measurements obtained in our study also revealed that the yoga position associated rise in IOP occurred within one minute after taking the position and that, in a similar manner, the IOP returned to the pre-exercise values within two minutes after being seated.

In previous studies, the evaluation of IOP and body position typically used a fixed measurement sequence. We used the Reichert Model 30 Pneumatonometer in a baseline, immediate, 2 minute, post, and 10 minute sequence for each position. This presents difficulties in interpretation because IOP measurements are affected by the measurement sequence. This applies due to multiple IOP measurements being taken in a short period of time and repeated measurements of IOP can result in a decrease in the readings. [24–26]

As a result of multiple IOP measurements, the magnitude of the changes owing to body position have been uncertain, with different studies reporting differences between sitting and supine IOP ranging from 0.3 to 5.6 mmHg for normal and glaucoma subjects; the use of the Mackey-Marg tonometer with calibration from a pneumatonograph and the Medtronic Model 30 Classic pneumatonometer were used for these data collections.[13, 27, 28] Through the asana (yoga position) change analysis we identified changes in IOP during four standard poses other than the previously studied sirsasana, in glaucoma and healthy control subjects. Inverted positions increase IOP significantly, but common positions have been incompletely investigated. Yoga practitioners may need to be aware of IOP changes during common yoga positions.

The yoga pose-associated rise in IOP may be explained by the hydrostatic increase in the pressure of episcleral veins and orbital veins into which aqueous humor is eventually drained and the pressures of which directly influence the IOP according to the Goldmann equation, Po = (F/C) + Pv, where Po is the IOP in mmHg, F is the rate of aqueous formation, C is the facility of outflow, and Pv is the episcleral venous pressure. Another factor which may potentially be involved in position-associated IOP changes may be changes in choroidal thickness. The choroid is drained through the vortex veins, which continue into the superior ophthalmic vein and finally into the intracranial cavernous sinus. Body position-associated changes in the intracranial cerebrospinal fluid pressure (CSFP) may thus indirectly influence the venous pressure in the choroid and the choroidal thickness and volume.[29]

Since elevated IOP is the most important known risk factor for development and progression of glaucomatous optic neuropathy, the rise in IOP after assuming the yoga poses is of concern for glaucoma patients. It has remained elusive whether the concomitant rise in cerebrospinal fluid pressure as the trans-lamina cribrosa counter-pressure against the IOP sufficiently compensates in amount in a timely manner for the rise in IOP. This study can therefore neither warn glaucoma patients not to perform yoga poses with head-down positions nor negate the possibility of exacerbating glaucomatous damage when performing yoga exercises with head-down positions.

Potential limitations of our study should be mentioned. First, the glaucoma group was significantly older than the non-glaucomatous group, so the comparison between both groups in the yoga pose-associated change in IOP should be cautiously interpreted. We minimized this effect by including age as a covariate in the multivariate analysis. Second, blood pressure was not measured; thus no information was obtained which could point to associated changes in cerebrospinal fluid pressure due to yoga position. Third, the duration of each pose was <5 minutes, therefore the study design does not allow conclusions on the change in IOP if yoga positions are kept for 30 minutes or an hour, such as in a formal yoga setting or class. Fourth, the number of study participants was relatively small, which could help explain the lack of statistically significant differences between the glaucoma and the non-glaucoma groups in the yoga associated IOP changes, and which does not necessarily suggest that there is no difference. Absence of proof is not necessarily a proof of absence if the study sample is small. Future studies with a larger study sample may be needed to further explore the association of the IOP changes in glaucoma and non-glaucoma groups. Fifth, in an attempt to keep a controlled order for all subjects, the order of poses was not randomized. A randomized order of poses could be analyzed in a future study. Such effects are likely to be minimal, as all IOPs went back to baseline before performing the next pose. The significance of variables such as age and body mass index should be interpreted with caution. Seventh, the design of our study did not allow examining changes in cerebrospinal fluid pressure (CSFP) during the yoga poses or examining a progression of glaucoma during the yoga poses and when yoga exercises are often performed. The purpose of our investigation was to assess whether, when and for which amount of IOP changes occur during yoga poses in normal individuals and in glaucoma patients. Despite the relatively small sample size, the results appear to be clear that in normal and in glaucoma patients, the IOP increases rapidly after taking the poses and re-normalizes rapidly as soon as the yoga exercises end. Future studies may now be warranted to further elucidate the interplay of IOP increase and the increase in CSFP and to assess whether yoga poses are a risk for glaucoma patients. Based on the results of the present study, one may state that glaucoma patients (as normal individuals) do experience an increase in IOP as soon as they hold a head-down yoga pose, and that that may be of potential risk for a glaucomatous optic nerve. The main value of the study is that it may have suggested in a qualitative manner as a proof of principle that the IOP rapidly adjusts to acute changes in body position. It raises new questions and potentially initiates larger-scaled studies on which parameters these IOP changes depend, including factors such as the speed of change in body position, duration of staying in the new body position, and associated changes in blood pressure, jugular vein pressure and in episcleral venous pressure.

In conclusion, normal subjects and open-angle glaucoma subjects experienced a statistically significant and rapid increase in IOP shortly after starting yoga exercises with head-down positions. In a similar manner, IOP dropped shortly after stopping the yoga exercises. Future studies may address whether the yoga pose associated rise in IOP markedly differs between glaucoma patients and normal individuals and if yoga practitioners performing these positions for a longer time period will have a longer duration of IOP rise. Although elevated IOP is a major risk factor for glaucomatous optic neuropathy, it remains unclear whether the yoga pose with head-down position associated with a rise in IOP increases the risk of progression among glaucoma patients.

## Supporting Information

S1 TREND Checklist TREND Checklist. (PDF)

S1 Protocol Protocol. (DOC)
