# Supplementary material for: Intraocular Pressure Rise in Subjects with and without Glaucoma during Four Common Yoga Positions
Source: PLoS One. 2015 Dec 23;10(12):e0144505. doi: 10.1371/journal.pone.0144505 (PMC4689525; doi:10.1371/journal.pone.0144505)
Supplement: S1 Protocol — (DOC) [file pone.0144505.s002.doc]

**TITLE: Intraocular pressure fluctuation in patients with glaucoma and in healthy patients during standard yoga positions**

**Primary Investigator:** Robert Ritch, MD

**Co-Investigators:** Jeffrey Liebmann, MD

Celso Tello, MD

Christopher Teng, MD

Jessica Jasien, MEn

Stephanie Muylaert, MD

**ABSTRACT:**

Glaucoma is the leading cause of irreversible blindness in the United States and can dramatically affect the quality of life for patients with moderate to severe visual loss. Glaucoma is characterized by a specific pattern of optic neuropathy and visual field loss secondary to death of retinal ganglion cells and represents the final common pathway of multiple diseases which affect the eye. Elevated intraocular pressure (IOP) is the most common known risk factor for glaucomatous damage and at the current time, the only modifiable risk factor for which treatment has been proven to have an effect on preventing or slowing the progress of the disease.

IOP increases on assuming a body position other than the upright one. ^(21,12,13,7,4)^ A small variation can be detected when moving from the sitting to recumbent position.^(2,22)^ The increase in IOP is directly related to the inclination of the body toward the complete inverted position.^(5)^ IOP begins to rise upon assuming a head down position and with the body vertical, results in doubling of the IOP,^(23)^ which remains elevated as long as this position is maintained.^(8,14)^

Postural yoga (asanas), including headstand posture (sirsasana), is, along with breathing exercises (pranayama) and meditation (dhyana), one of the three basic components of hatha yoga. Yoga has become a popular practice in the western world. In 1998 an estimated 15 million American adults had performed yoga at least once in their lifetime.^(3)^ Recent studies have described an elevation in IOP following sirsasana (headstand) posture, particularly in glaucoma patients.^(3,9)^ In the sirsasana position there was a uniform 2-fold increase in IOP.^(3,9,15)^

Those who have been practicing yoga for many years may present with glaucomatous optic atrophy and visual field defects despite a normal IOP when measured in the sitting position. This may lead to diagnosis of normal tension glaucoma. The term “intermittent glaucoma” was suggested by Schuman et al in their investigation of high resistance wind instrument players, ^(19)^ and may be also used for those who practice inverted positions during yoga and otherwise exhibit normal intraocular pressures at office visits.

Previous studies evaluating IOP and body position have typically used a fixed measurement sequence. This presents difficulties in interpretation because IOP measurements are affected by the measurement sequence,^(16)^ and repeated measurements of IOP can result in a decrease in the readings.^(1, 10)^ As a result, the magnitude of the changes owing to body position have been uncertain, with different studies reporting differences between sitting and supine IOP ranging from 0.3 to 5.6 mmHg for normal and glaucoma subjects ^(23,11,20)^.

We plan to use standard ophthalmic tonometry devices to monitor IOP changes during different standard yoga asanas. An example of a standard yoga asana is the adho mukha svanasana, which is most commonly known as the downward facing dog pose. This asana is performed by kneeling on all fours with hands beneath the shoulders and the knees directly beneath the hips. The hips should be hip width apart and the fingers spread wide apart. Tuck the toes under as inhaling and lifting the body up, keeping the shoulders back and pushing backward on the hips, keeping the heels down as much as possible. Exhale and relax the neck muscles while lowering the head to look back at the feet.^(17)^ This is shown in Figure 1.


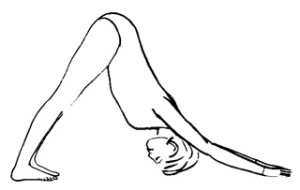


Figure 1. Adho Mukha Svanasana.^(17)^

Another example of a common asana is the uttanasana, which is most commonly known as the standard forward bend pose. This asana is performed by resting the hands on the hips, exhaling and bending forward from the hip joints. By keeping the knees straight, place the fingertips or palms on the floor beside the feet or touching the back of the ankles with the palms (this is shown in Figure 2). This can also be modified by crossing the forearms and holding the elbows (this is shown in Figure 3). Allow the head to hang loose and release all the tension in the back and shoulder blades.^(17)^


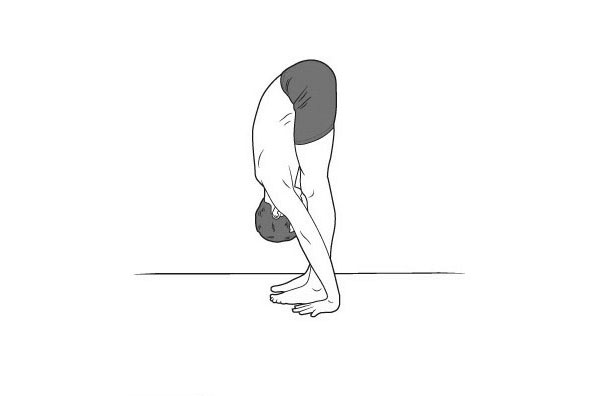


Figure 2. Uttanasana with palms on the floor.^(17)^


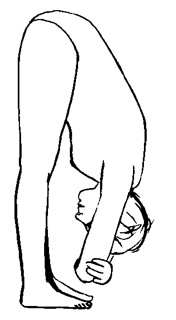


Figure 3. Uttanasana modified.^(17)^

Through the asana change analysis we aim to further our understanding of the differentiation of IOP during standard yoga asanas of glaucoma and healthy participants. To the best of our knowledge, there have been no studies on the IOP variation with asanas, other than Sirsasana.

**DISCUSSION:**

**A) Specific Aims**

We plan to identify the IOP difference between normal and glaucomatous eyes using standard ophthalmic tonometry devices while subjects are performing standard yoga asanas. The IOP of participants will be measured prior, immediately at start of the asana, 5 minutes into the asana, and immediately after assuming a sitting position. The patient will wait 10 minutes and a final IOP will be taken.

**Study Design**

Participants will be identified and recruited from 1) patients at Glaucoma Associates of New York who are under the care of Drs. Robert Ritch, Jeffrey Liebmann, Celso Tello, and Christopher Teng; 2) through yoga centers in New York; and through yoga instructors who are patients of the practice. A total of 20 patients will be tested – 10 glaucoma patients and 10 control patients.

Technical and medical staff, under the supervision of Dr. Ritch, will aid participants in the completion of standard yoga asanas.

Data will be de-identified. De-identified data may be retained by the Investigators for analyzing purposes. The clinical coordinator of the project will oversee involvement of other staff for the procedures.

**Study Procedures**

Ophthalmologists will:

1. Identify subjects
2. Demographics, medical and ophthalmic history, glaucoma family history and ocular findings will be recorded for each study participant.
3. Obtain informed consent.
4. Collection of data will be obtained at Glaucoma Associates of New York.
5. Participants will have undergone a full eye examination; including refraction, slit-lamp examination, gonioscopy, visual fields, and optic disc exam prior to entry into the study.
6. The selected Yoga asana, for example adho mukha svanasana (Figure 1) or uttanasana (Figure 2) to name two, will be held for 5 minutes. Measurements of IOP will be collected using standard ophthalmic tonometry devices at set time points.
7. IOP taken OU prior to asana with tonometry.
8. Participant begins asana. A second IOP measurement is taken immediately after assuming the asana.
9. Five minutes after assuming the asana, a third IOP measurement is taken. Participant can then resume normal seated position.
10. Immediately assuming seated position, a fourth IOP measurement is taken.
11. 10 minutes after seated position, a final IOP is taken.
12. The study procedures will then be repeated in a different standard yoga asana.
13. De-identify and code data. All data files will be de-identified, with the patient's identifiers removed from the sample and a unique code assigned.

**B) NATURE OF CONTACT WITH HUMAN SUBJECTS**

1. Level of Risk for patients

__x__ _Minimal Risk

______ Minor Increase over Minimal Risk

______ More than Minor Increase over Minimal Risk

1. Potential Risks Include: A rise in IOP is expected but will be transient and not expected to be harmful.
2. Procedures to Minimize Risk: Routine Precautions

**C) SUBJECT ENROLLMENT**

Volunteers will be recruited from the patients at private offices of ophthalmologists. Persons between ages 18 – 80 will be entered. All participants must be familiar with standard yoga asanas. Participation is open to all who meet the diagnosis and age requirements.

**Inclusion Criteria**

1. Written informed consent.
2. Age 18 to 80 years
3. Glaucoma patients or healthy controls with normal examination.
4. Yoga practitioner, someone who has been performing yoga practice for more than 12 months. Participant must also be able to maintain standard yoga asanas for a minimum of five minutes.

**Exclusion Criteria**

1. Unwilling to sign informed consent.

2. Participant cannot maintain standard yoga asanas for a minimum of five minutes.

3. Healthy subject does not have a normal ophthalmic examination.

Participants will not be reimbursed for participation in the study.

**Confidentiality**

Dr. Ritch's group will maintain a code protecting patient confidentiality.

**Duration of project and total amount of time required of each subject.**

Subject involvement is limited to the time of recruitment and completion of standard yoga asanas and IOP measurements. Recruitment (including patient education, questions, reading of the Informed Consent Form) is expected to take approximately 20 minutes. The time involvement for patients to complete the asanas and have their IOP taken will be about an hour of their time.

**D) SAFETY:** Medical staff and personnel will be with the participants during the

length of study time. All asanas will be performed by yoga practitioners. Risk of psychosocial harm to any kindred or group is none.

**E) RADIATION:** Not applicable

**F) DEBRIEFING:** Results from research will be de-identified and will only be used for research purposes. Any reference to the data from this point on for research purposes, including publication, is made by study identification number only. If, in the future, more concrete information about participant’s diagnosis becomes available from this study, the patient’s ophthalmologist will inform the patient.

**G) RISK/DECEPTION:** Physical, psychological, social, legal, economic, or any other risk. The anticipated risks associated with this study are those associated with the yoga asana.

**Justify conduction of "non-beneficial research"** (i.e. research involving investigation of a person, his/her body, life, or surroundings, which has no benefit to that person.)

The purpose of completing this asana is beneficial to all glaucoma and healthy patients who practice yoga. Studies have only been performed on headstand posture; a head down posture may also cause a significant rise in IOP. The testing of standard yoga asanas will help show the change in IOP in the asana. The practice of yoga could be dangerous for all yoga practitioners, especially glaucoma patients due to the change in IOP.

**H) CONFIDENTIALITY OF SUBJECTS:** The data of this research and summaries of medical histories may be used in publications, presentations, and grant or funding applications. No personal identifiers will be disclosed. In the unlikely event that an outside agency requests access to the data, the data would only be made available via requests approved by New York Eye and Ear Infirmary IRB.

Dr. Ritch's group will maintain possession of the signed Informed Consent Forms.

**REFERENCES ALPHABETICAL**

1. Almubrad TM, Ogbuehi KC. On repeated corneal applanation with the Goldmann and two non-contact tonometers. Clin Exp Optometry 2010; 93:77– 82.
2. Anderson DR GW. The influence of position on intraocular pressure. IO 1973; 12:204-212.
3. Baskaran M, Raman K, Ramani KK, Roy J, Vijaya L, Badrinath S. Intraocular pressure changes and ocular biometry during Sirsasana (headstand posture) in Yoga practitioners. Ophthalmology 2006; 161:1327-1332.
4. Buchanan RA WT. Intraocular pressure, ocular pressure, and body position. Am J Optometry physiological Optics 1985; 62:59-62.
5. Carlson KH MJ , Tpper JE , Brubaker RF.  Effect of body position on intraocular pressure and aqueous outflow. IOS 1987; 28: 1346-1352.
6. Fahmy JA, Fledelius H. Yoga-induced attacks of acute glaucoma. A case report. Acta Ophthalmology 1973; 51:80-84.
7. Friberg TR WR. Ocular manifestations of gravity inversion. JAMA 1985; 253:1755-1757.
8. Friberg TR SG. Optic nerve dysfunction during gravity inversion. Pattern reversal visual evoked potentials. Arch Ophthalmology 1985;103: 1687-1689.
9. Gallardo M, Aggarwal N, Cavanagh HD, Whitson JT. Progression of Glaucoma Associated with the Sirasana (Headstand) Yoga Posture. Advances in Therapy 2006; 23, 6: 921-925.
10. Gaton DD, Ehrenberg M, Lusky M, et al. Effect of repeated applanation tonometry on the accuracy of intraocular pressure measurements. Curr Eye Res 2010; 35:475–9.
11. Jain MR, Marmion VJ. Rapid pneumatic and Mackey-Marg applanation tonometry to evaluate the postural effect on intraocular pressure. Br J Ophthalmology 1976; 60:687–93.
12. Klatz RM GR, Pinchuk BG, Nelson KE, Tarr RS. The effects of gravity inversion procedures on systemic blood pressure. J Am Optometric Association 1983; 82:853-857.
13. LeMarr JD GL, Adler JG. Intraocular pressure response to inversion. American Journal of optometry and physiological optics 1984; 61:679-682.
14. Linder BJTG, Wolf ML. Altering body position affects intraocular pressure and visual function. Invest Ophthalmology Visual Science 1988; 29:1492-1497.
15. Monteriro de Barros DS, Bazzaz S, Gheith ME, Siam GA, Moster MR. Progressive optic neuropathy in congenital glaucoma associated with the Sirasana yoga posture. Wills Eye Institute Papers 2008; 3.
16. Pekmezci M, Chang ST, Wilson BS, et al. Effect of measurement order between right and left eyes on intraocular pressure measurement. Arch Ophthalmology 2011; 129:276–81.
17. Pettinato, Yolando. Simply Yoga. Hinkler Books 2002; 22-23.
18. Rice R, Allen RC. Yoga in glaucoma. Am J Ophthalmology 1985; 100(5):738-739.
19. Schuman JS ME, Connolly S, Hertzmark E, Mukherji, Kumen MZ. Increased intraocular pressure and visual field defects in high resistance wind instrument players. Ophthalmology 2000; 107:127-33.
20. Sit AJ, Nau CB, McLaren JW, et al. Circadian variation of aqueous dynamics in young healthy adults. Invest Ophthalmology Visual Science 2008; 49:1473–9.
21. Tarkkanen ALJ. Positional variations of the intraocular pressure as measured with Mackay-Marg tonometer. Acta Ophthalmology Scand 1967; 45:569-575.
22. Weber AK PJ. Pressure differential of intraocular pressure measured between supine and sitting position. Annals 1981:13:323-327.
23. Weinreb RN CJ Friberg, TR, Effect of inverted body position on intraocular pressure. Am J Ophthalmology 1984; 98:784-787.
